# The Book World of Medicine and Science

**Published:** 1911-09-23

**Authors:** 


### The Book World of Medicine and Science.

Physiology of the Central Nervous System and the Special Senses. By N. J. Vazifdar, L.M. & S. (Bombay: James and Sons. 1911.)

Originally written for students in the Inter. M.B. classes of the Bombay University, these notes have now been published in order to reach a wider class of students. The book consists of a fairly complete digest of the physiology and the anatomy of the nerve tracts of the central nervous system, with chapters on the special senses. Eccentricities in printing and numerous errors in English and Latin do not inspire great confidence, but the book is a praiseworthy attempt to tabulate and summarise the large amount of detail necessarily associated with the study of the central nervous system.

Meat and Food Inspectors' Examinations: Model Answers to Questions set by Examining Bodies. By G. T. Billing and A. H. Walker. (London: The Sanitary Publishing Co., Ltd. Pp. 156. 1911. Price 3s. 6d. net.)

It is well recognised that one of the best ways of ensuring success at an examination is to study carefully the questions asked on previous occasions. This is generally easy enough in the case of the papers to be written, but it is not so often possible to gauge accurately the scope of questions asked in the *viva voce* and practical portions of examinations. The value of this book is decidedly enhanced by the inclusion of a large number of *viva voce* questions asked at the "theory" table and at the "practical" table, where specimens of meat, fish, bones, etc., are displayed. The book, which gives full information concerning the meat and food inspectors' examination, covers the whole syllabus of that particular examination, and will also be found of great value to candidates for the examinations of the Board of Education in hygiene, of the Royal Sanitary Institute, and of the Sanitary Inspectors' Board. We can also recommend it to the attention of candidates for a diploma in public health. The many and varied subjects of the syllabus are well represented in the 150 questions selected from recent papers and the answers given form an excellent course of instruction. The book is ably and carefully prepared, and we have no hesitation in saying that it deserves a large measure of popularity among intending candidates for meat inspectorships on account of its helpful and practical character.

Personal Hygiene and Physical Training for Women. By Anna M. Galbraith, M.D. Illustrated. (Published in Philadelphia and London. 1911. Price 10s. net.)

If it be true that women of all ages take themselves and life too seriously, it is certain that in this discursive manual womankind is taken very seriously indeed by the author. She is evidently in favour of the maxim of Herbert, "Who aimeth at the sky, shoots higher much than he that means a tree," and consequently treats the subject of personal hygiene not only fully but from a lofty hygienic pinnacle. The first chapter deals with the application of water, both internally and externally, for health purposes, and contains descriptions of numerous modifications of the ordinary bath, and the douche in all its variations. The skin and its appendages are naturally of prime importance and interest to the female sex, so we find an exhaustive chapter is devoted to this section. We must admire the thoroughness and the earnestness with which Dr. Galbraith has endeavoured to instruct her sex in the important physiological principles underlying all attempts at hygienic living, but we doubt the usefulness of including so much as is to be found in this book. The insertion of complex prescriptions which contain powerful drugs is not a wise thing, seeing that the work is intended for lay women; the proportion of resorcin, for instance, in one recipe is unnecessarily large.

After excellent chapters on the digestive and other systems, we come to the nervous system, and here we have an interesting essay on work, rest, worry, fatigue. In many cases the author's recommendations must be, for the ordinary woman, mere counsels of perfection. We wonder how many readers would undress completely and go to bed for an hour's nap in the afternoon, or carry out a daily douching of the nose. Much philosophic sentiment is included in the chapter on the hygiene of the mind, and the author rises to a dissertation on the psychology of success.

The more practical portion of the book—that devoted to physical training—is encumbered with bits of physiology and mythology, but is adorned with excellent plates of certain classical sculptures. The description of various dances and gymnastic exercises and other means of attaining and maintaining a graceful and healthy physique, has the advantage of a large number of plates illustrating fully the different positions and movements.

This volume is obviously one containing information and instruction of the highest importance to women, and much advice that could profitably be followed, but the value of the work is unfortunately diminished by a deal of redundant verbiage which obscures the good things, and there is also a certain amount of advice which is of questionable utility or of which the *rationale* is founded on unsupported premisses. These defects are not irremediable, and the book has so much of excellence in it that we may express a hope that there may one day be produced for English readers an edition revised in this respect and with such expressions as "sorbefacient of pathologic products" deleted therefrom.

The Life-History, Function, and Inflammation of the Appendix. By E. M. Corner, M.C., F.R.C.S. (London: John Bale, Sons, and Danielsson. 1911. Pp. 23. Price 1s. net.)

In this essay Mr. Corner propounds a speculation originally advanced by Mr. Battle, concerning the origin of appendicitis. The gist of the author's indictment lies on pages 10 and 11, where he explains the coincidence between the first epidemic of appendicitis in America twenty years ago and the contemporaneous invention of steel rollers for preparing flour in the mills over there. He points out that these steel rollers rapidly become worn down and have to be replaced by new ones. The inference drawn is that the steel particles must be in the flour thus ground, and Mr. Corner thinks that these particles may be one—but not necessarily the only one—of the causes for the prevalence of appendicitis to-day. He states that appendicitis became common in England about the same time that American steel ground flour was first imported in large quantities; and that in the United States the blacks, who for a long time clung to their old-fashioned stone-mill flour, had a marked freedom from appendicitis for the first few years of its incidence upon the white population. Finally the stone mills had to shut down by the fierce competition, and since then the negroes have had appendicitis as frequently as others in America. The hypothesis may or may not be right; but at least it demands investigation.

A Pocket Atlas and Text-book of the Fundus Oculi. By G. L. Johnson, M.D., F.R.C.S., and Arthur W. Head, F.Z.S. (London: Adlard and Son, Bartholomew Close, E.C. Price 10s. 6d. net.)

This handy volume should prove of distinct service to senior students and graduate workers in ophthalmology. Essentially a practical manual, it gives very clear instructions regarding the manner in which the ophthalmoscope should be used and excellent hints on the best types of instrument to obtain. Following this comes a series of short and admirably condensed sections dealing with the various phases of retinoscopy, errors of refraction, and diseases of the eye generally. The Appendix, for example, is a most useful summary of clinical hints and practical points in which the various ophthalmic reactions are fully discussed, although the short section concerning "606" is no longer quite up to date. At the end of the book are a number of drawings of the fundus, both in normal and in abnormal conditions, while a thoroughly practical ophthalmic note and drawing book, which ought to appeal to clinicals in the eye departments, is inserted at the end. We cordially commend the work to the attention of the general practitioner. It is an accurate, handy, and reliable manual, and may be regarded as a good investment by the student or practitioner who wishes to have on his shelves an epitome of ophthalmoscopy. The drawings of the fundus are especially clear, and are perhaps the best of their kind to be found in English text-books.

Essentials of Medicine. By Charles Phillips Emerson, M.D. Second edition, revised. (J. B. Lippincott Co. 1911. Pp. 401. Price 8s. 6d. net.)

As we reviewed this book on its first appearance two years ago, we need do little more than call attention to the new edition. The scope and substance of the book remain practically the same as before. But since 1909 the Flexner report has thrown a flood of light upon the American medical schools and their methods. One is able, therefore, now to understand much more fully why such a book as this should be offered to American medical students, and to comprehend that it gives them a perspective which they probably do not get (in many of the less efficient schools) during their ordinary curriculum and training. In the attempt to put forward a very elementary and very much simplified account of medicine, which shall yet have a rational philosophical basis and shall eliminate the rarities and curiosities, the author has well succeeded. That such a book should be needed at all by medical students shows a radical defect, we think, in the educational system under which they are brought up, and goes far to confirm and to explain the revelations of the Carnegie Report. For nurses and for the most intelligent section of the lay public the book is obviously an attractive one; for medical students it should be superfluous. These opinions, formed on first acquaintance with the book, we still retain; but we now perceive, thanks to Dr. Flexner, the reason why in the United States the medical student needs assistance in the orientation of his ideas such as we still hope and believe is not necessary for his counterpart in Great Britain.

Bone Setting and the Treatment of Painful Joints. By F. Romer, M.R.C.S., L.R.C.P. and L. E. Creasy, M.R.C.S., L.R.C.P. (London: James Nisbet and Co. 1911. Price 1s. net.)

Notwithstanding its ambitious title, this small volume contains nothing that is not likely to be perfectly well known to a senior dresser in the surgical wards, while it offers certain statements with which expert bone-setters will hardly agree. When the authors have had more experience of bone setting they will probably alter some of the curious opinions expressed—such as for instance that gas is not a suitable anæsthetic for manipulative treatment. The book certainly does not "dispel the notion that the medical profession knows nothing about bone setting."

A Synopsis of Surgery. By E. W. Hey Groves, M.D., M.S., F.R.C.S. Third edition. (Bristol: John Wright and Sons, Ltd. 1911. Price 9s. 6d. net.)

When the first edition appeared in 1908 we expressed the opinion that this book was likely to become exceedingly popular with students owing to the great care which has been taken in ensuring accuracy of statement and simplicity of arrangement. Since then the book has run into two editions, and this, the third, is a marked advance upon its predecessors. The various chapters have been systematically revised and brought up to date, and students may feel confident that, armed with this synopsis, they have at hand an epitome of the latest and most reliable data on surgical topics. Whether such condensations are altogether to be commended to the student is another question. In view of the present day tendency to lay undue stress upon the results obtained by examination, the use of such compilations is perhaps excusable, and in any case Mr. Groves' attempt to epitomise modern surgical teaching is the best of its kind that we have yet seen. Each subject is dealt with in a succinct and more or less dogmatic fashion, as is indeed advisable in such a condensation; the differential diagnosis is especially well treated in each case, while new methods are outlined with commendable clearness, as is evidenced by the admirable résumé of modern methods of attacking the lung on page 344. The teaching is throughout accurate, and no exception can be taken to the various statements made, since in each case alternative points, opinions, and methods are given. Altogether it is a book which should prove of great service to the student who is revising his work for the Final in surgery.

